# Effect of leaf type on browse selection by free-ranging goats in a southern African savanna

**DOI:** 10.1371/journal.pone.0242231

**Published:** 2020-11-11

**Authors:** Casper C. Nyamukanza, Allan Sebata

**Affiliations:** 1 Department of Animal and Wildlife Sciences, Midlands State University, Gweru, Zimbabwe; 2 Department of Forest Resources and Wildlife Management, National University of Science and Technology, Bulawayo, Zimbabwe; Institute for Biological Research "S. Stanković", University of Belgrade, SERBIA

## Abstract

Broad- and fine-leaved woody species respond to seasonal changes from wet to dry season differently. For example, broad-leaved species shed their leaves earlier, while fine-leaved species, especially acacias retain green foliage well into the dry season. These differences are expected to result in variation in selection of broad- and fine-leaved woody species as browse by free-ranging goats. We tested the hypothesis that free-ranging goats select broad-leaved woody species more than fine-leaved species during wet (growth) season and fine-leaved woody species more than broad-leaved species during dry season. In addition, we tested if broad- and fine-leaved woody species had different foliar dry matter digestibility and chemical composition (crude protein, neutral detergent fibre, acid detergent fibre, total phenolics and condensed tannins concentration). Free-ranging goats were observed foraging on broad- and fine-leaved woody species over a two-year period (2014 and 2015) during three seasons: early wet (October/November), late wet (February/March) and dry (May/June). Ivlev’s selectivity or Jacob’s index (*E*_*i*_) was calculated for five woody species (two broad-leaved and three fine-leaved) browsed by goats during wet and dry season. Jacob’s selectivity index was higher for broad–leaved (*Ziziphus mucronata* and *Searsia (Rhus) tenuinervis*) than fine-leaved woody species (*Acacia nilotica*, *Acacia karroo* and *Dichrostachys cinerea*) during wet season. However, the trend was reversed during dry season with fine-leaved species having higher Jacob’s selectivity index than broad-leaved species. Leaf dry matter digestibility and chemical composition was similar between broad- and fine-leaved woody species throughout the year. We conclude that goats selected broad-leaved woody species during wet season when browse was plentiful and then switched to fine-leaved species which retained leaves during dry season.

## Introduction

Woody species play an important role as browse for goats in semi-arid and arid savanna ecosystems in southern Africa. Semi-arid and arid savanna ecosystems are characterized by distinct wet and dry seasons. The transition from wet to dry season or *vice versa* is associated with loss and production of new tree leaves, respectively [1]. Therefore, browse is readily available in wet (growth) season and becomes scarce during dry season as woody species shed their leaves [2,3]. Both broad- and fine-leaved woody species mostly sprout during wet season and shed leaves during dry season. However, broad-leaved woody species shed almost all their leaves during dry season [4], while fine-leaved species, especially acacias largely retain green foliage well into the dry season [3]. Goats as opportunistic feeders are expected to exploit the contrasting leaf phenology patterns of broad- and fine-leaved woody species. Mellado [5] reported goats as effectively exploiting forage resources in rangelands through their flexible, broad-scale and opportunistic behaviour. Thus, goats should select broad-leaved species more during wet season to exploit their higher foliar mass than fine-leaved species [4], and then switch to fine-leaved woody species that retain most of their leaves during dry season [6]. This prediction need to be tested because foraging habits of goats are highly variable particularly in response to ecological and seasonal variation in browse availability and quality [7,8].

Goats actively select plant parts and species to consume, implying that their foraging is not a random process [9]. Goat browse selection is mainly considered to be driven by foliar nutritive value and anti-nutritional factors [10]. For instance, goats select parts of plant species with high nutritive value in terms of high crude protein (CP) content and digestibility [11,12], low fibre content [13], and low phenolics [12], to maximize nutrient intake [14] and minimize anti-nutrient intake [15]. However, when browse resources in the rangeland are of similar nutritive quality, goats are expected to use other selection criteria in their forage choices. The nutritive value of broad- and fine-leaved woody species has not been compared limiting our ability to understand goat foraging choices on these important browse species. Woody plant leaf chemical components such as CP, fibre (neutral and acid detergent fibre), total phenolics (TP) and condensed tannins (CT) concentrations are important proxies for browse nutritive value [6,10,15–18]. Dry matter digestibility (DMD) is also a good proxy for browse nutritive value as it is a measure of forage energy value [10]. However, use of leaf chemical components to predict browse nutritive value is not always conclusive [19]. Crowell et al. [20] suggested that CP and fibre were poor predictors of nutritive value of browse of generalist browsers. Crude protein content of most woody species is generally within a narrow range [4], presumably contributing to it being a poor predictor of browse nutritive value for goats. However, Mphinyane et al. [8] reported a positive relationship between CP and browse nutritive value.

High foliar fibre content negatively affects browse nutritive value [9,21,22], because it constrains digestibility [23]. Hence, foliar fibre content (both neutral and acid detergent fibre) can be used to predict browse nutritive value for goats [9]. Similarly, browse with low phenolics content [24], is expected to have high nutritive value as it does not reduce digestibility of browse through inhibiting microbial action in the gastrointestinal tract [25]. Condensed tannins also affect browse nutritive value and in turn reduce its intake thereby negatively affecting animal performance [26]. However, condensed tannins are considered beneficial at levels below 5% of diet as they improve utilization of forage [27], and also reduce internal parasitic loads [28] and methane emissions [29]. Interestingly, Mellado [5] found no evidence that browse choice by goats was biased towards plants with low secondary compounds.

The objective of this study was to determine if goats select broad- and fine-leaved woody species differently during wet and dry season. In addition, we determined if broad- and fine-leaved woody species had different foliar dry matter digestibility (DMD) and chemical composition. We hypothesized that: (i) free-ranging goats select broad-leaved more than fine-leaved woody species during wet (growth) season and fine-leaved more than broad-leaved woody species during dry season, (ii) broad- and fine-leaved woody species have different foliar DMD and chemical composition [CP, neutral detergent fibre (NDF), acid detergent fibre (ADF), TP and CT concentration].

## Materials and methods

### Experimental area

**Fig 1:**
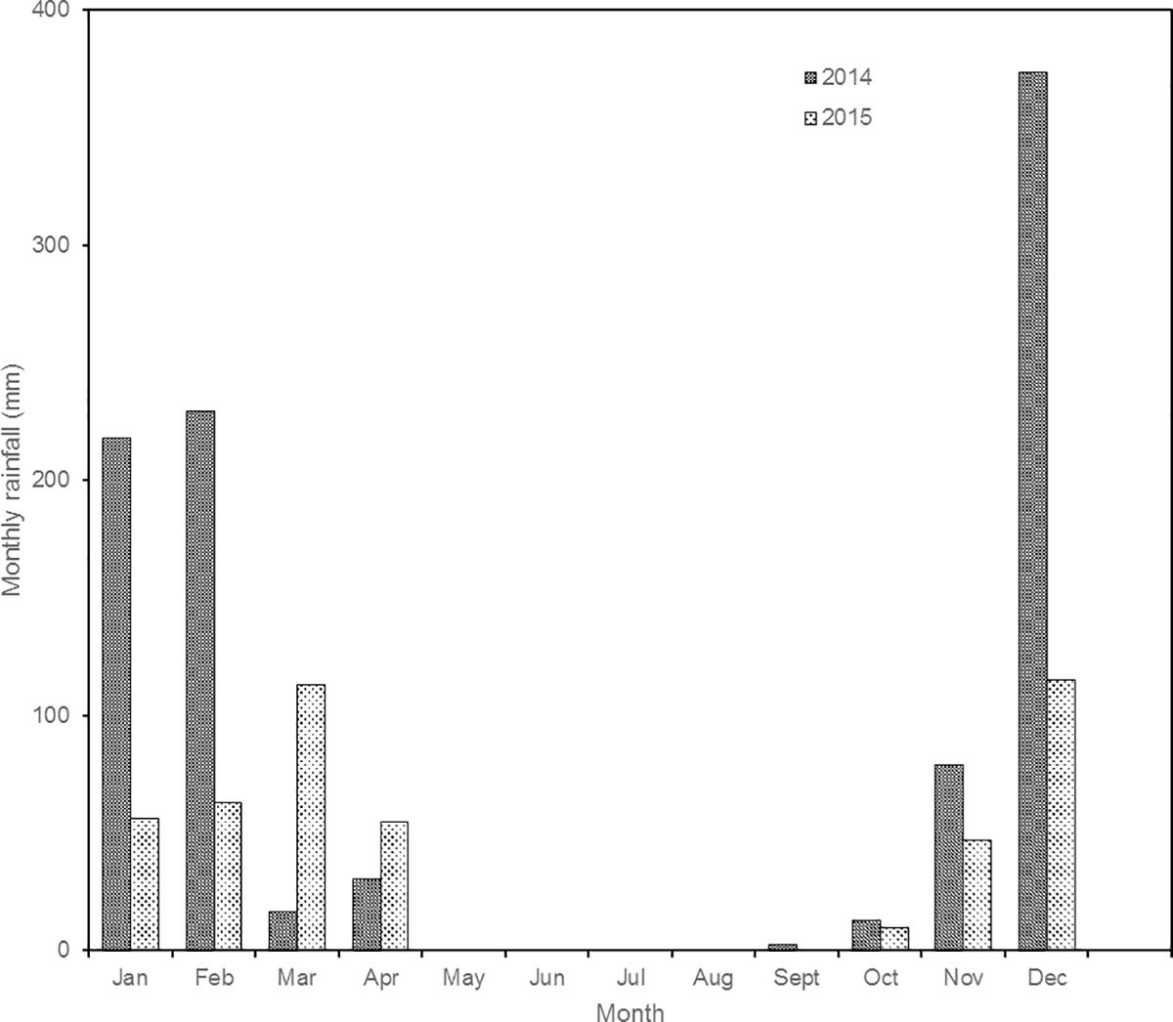
Total monthly rainfall at Gweru Thornhill meteorological station for 2014 and 2015 (Source: Meteorological Services Department of Zimbabwe).

The study was conducted for two consecutive years (2014 and 2015) at Riversdale newly resettled farming area (19°14'S; 29°40'E; 1360m above sea level), a 30ha farm 25 kilometers northwest of Gweru, central Zimbabwe. The annual rainfall was 961.9mm and 458.4 mm for 2014 and 2015 respectively (Fig 1), with a long term mean annual rainfall of 700mm, 80% received between November and April, recorded over the last 50 years. For both January-February and November-December periods, rainfall was higher in 2014 than in 2015 (447.4 and 452.2 against 119 and 161.9 mm, respectively) (Fig 1). Average minimum temperature in July was 12.8°C and maximum temperature in October was 21.5°C. The vegetation type is open southern Miombo woodland dominated by *Brachystegia spiciformis* and *Julbernadia globiflora* with *Acacia nilotica*, *Acacia karroo*, *Dichrostachys cinerea*, *Searsia tenuinervis* and *Ziziphus mucronata* as the main browse species. The most abundant grasses are of the genera *Hyparrhenia* and *Andropogon*.

#### Relative abundance of browse species

Relative abundance of woody species in the rangeland was determined in ten 50m x 30m quadrats in March 2014. In each quadrat woody plants were identified to species level according to Coates-Palgrave [30] and van Wyk and van Wyk [31] and the abundance of the woody species recorded. Each woody species was expressed as a percentage calculated as: Total number of browseable plants of each species /Total number of all browseable woody plants in the rangeland x 100 [5].

*Acacia nilotica*, *A*. *karroo*, *D*. *cinerea*, *S*. *tenuinervis* and *Z*. *mucronata* were the most important browse species for the goats. Both *A*. *nilotica* and *A*. *karroo* are characterized by bipinnate leaves with tiny leaflets mainly on old shoots, are deciduous and have long straight thorns [30]. *Dichrostachys cinerea* (L.) Wight & Arn. ssp. *africana* also has bipinnate leaves with tiny leaflets borne on both new and old shoots, are deciduous and have long straight thorns of relatively low density [31]. *Searsia tenuinervis* is a deciduous species that has broad leaves and grows new leaves on new shoots; the shoots are spineless [31]. *Ziziphus mucronata* Willd. subsp. mucronata has broad leaves borne mainly on new shoots, is deciduous and has both hooked and straight thorns [31]. *Z*. *mucronata* is an important browse species for many browsers [32].

#### Experimental flock and its management

Small east African Mashona goats from four households were combined to form a flock of between 24 and 47 for the woody plant selection study during a two year (2014–2015) period. The flock comprised of mature lactating and non-lactating does, bucks and young stock. The goats from the four households were kept together for at least four weeks before observations to get used to each other. Goats were allowed to browse during the day and penned overnight. In the morning the goats were moved out of their housing and allowed to freely range in the natural rangeland during the day. A week before the study goats were subjected to a period of habituation to human presence resulting in the goats being approached to within 5 m without being disturbed. Ethical approval was obtained from Midlands State University Research Board Ethics Committee, approval number 2013/AWS0103.

#### Proportion of use of each woody species

Proportion of use of each woody species was estimated by direct observation of free-ranging goats whilst browsing. The free-ranging goats mostly foraged on woody plants. Each day, two different non-lactating adult female goats were randomly selected from 16–18 non-lactating females in the flock for observation, allowed to forage with others while being continuously observed from a distance of about 5m with minimal disturbance. Observations were made twice a day for seven consecutive days in October/November (early wet season), February/March (late wet season) and May/June (dry season). Direct observation of goats results in shorter distances between observer and focal animal and is very efficient in determining browse species consumed [33]. Each goat was observed foraging on rangelands in the morning from 09:00 to 11:00 h and in the afternoon from 12:00 h onwards until observations had been collected for at least 4 h per day for seven consecutive days. Duration of each feeding bout and species of woody plant from which bites were cropped was recorded. Feeding bout was defined as a 5-second period during which an animal fed continuously on a specific plant species. If the feeding bout lasted less than 5 seconds, the plant species was considered to be avoided. Proportion of use of each woody species was calculated as: total time spent on a woody species / total time spent on all species for each observation day. Species on which goats spent less than five seconds were not included in the calculation of proportion of use.

#### Jacob’s selectivity index

Ivlev’s selectivity or Jacob’s index (*E*_*i*_) was used to calculate the selection of each woody species using the formula: *E*_*i*_ *=* (*U*_*i*_*—A*_*i*_) */* (*Ui—Ai*), where *U*_*i*_ represents the proportion of use of woody species *i* and *A*_*i*_ its proportional availability in the rangeland [34]. The Jacob’s index was selected because it is simple and has been reported to be comparable with more complex methods [6]. Jacob’s selectivity index factors the differences in the relative abundance of feed resources when evaluating diet selection [35]. The selectivity index (*E*_*i*_) varies from -1 (indicating lower use relative to availability of woody species) to +1 (indicating higher use relative to availability of woody species) and a zero value indicating proportional use of woody species in relation to availability [36].

#### Leaf chemical analysis

Leaf material from each of the browsed species was collected for chemical analysis. The leaf samples were taken from five unbrowsed trees (below 1.5 m above ground) per species (*A*. *nilotica*, *A*. *karroo*, *D*. *cinerea*, *S*. *tenuinervis* and *Z*. *mucronata*). The leaf samples were air dried prior to oven drying at 60°C for 48 h. The leaf samples were then milled to pass through a 1.0 mm mesh using a Wiley mill and then analysed for CP, fibre (acid and neutral detergent fibre), TP and CT concentration. Each chemical component was expressed as a percent of the sample dry matter. Nitrogen (N) concentration was determined using the Kjeldahl method [37] and converted to crude protein (N x 6.25). Neutral detergent fibre and ADF were determined according to van Soest et al. [38] using the ANKOM Technology. Phenolic compounds were extracted and TP and CT concentrations determined. Total phenolic concentration was determined using the Folin-Ciocalteau method [39]. Gallic acid (0.5mg/ml) was used as a standard and the TP concentration was expressed as gallic acid equivalents (GAE). Condensed tannins concentration was determined using the butanol-HCl method and expressed as leucocyanidin equivalent [38]. Dry matter digestibility was estimated using the formula: DMD% = 83.58–0.824 ADF % + 2.626 N% [40].

#### Statistical analysis

During each season browse selection observations were made for seven consecutive days with two goats selected randomly for observation each day. Each goat woody (browse) species selection was treated as an independent observation during all seasons in the two years of the study because it was only used once for observations throughout the experimental period. We tested the effect of year, season, species, day and individual goat on Jacob’s selectivity index using a General Linear Model (GLM) univariate analysis of variance. A GLM univariate analysis of variance was also used to test the effect of year, season and species on leaf dry matter digestibility and chemical composition. Woody species Jacob’s selectivity index differences within and among the seasons were tested using one-way analysis of variance with Tukey *post-hoc* test used for pairwise comparisons. In addition, differences among seasons in dry matter digestibility and chemical composition of the five woody species were tested using one-way analysis of variance. Jacob’s selectivity index, leaf dry matter digestibility and chemical composition data for broad- and fine-leaved species were pooled and differences tested using Independent *t-*test using IBM SPSS Statistics 20.0 [41].

## Results

General linear model univariate analysis of variance showed that year (*F*_1,1_ = 0.01, *p* = 0.92), day (*F*_1,6_ = 0.04, *p* = 1.00) and individual goat (*F*_1,13_ = 0.03, *p* = 1.00) had no significant effect, while season (*F*_1,2_ = 7.82, *p* < 0.001) and species (*F*_1,4_ = 932.40, *p*< 0.001) had significant effect on Jacob’s selectivity index. Year had no significant effect on leaf dry matter digestibility (*F*_1,148_ = 3.19, *p* = 0.09), crude protein (*F*_1,148_ = 0.31, *p* = 0.59), neutral detergent fibre (*F*_1,148_ = 1.60, *p* = 0.22), acid detergent fibre (*F*_1,148_ = 3.13, *p* = 0.09), total phenolics (*F*_1,148_ = 1.03, *p* = 0.0.32) and condensed tannins (*F*_1,148_ = 0.87, *p* = 0.36). Season had a significant effect on leaf dry matter digestibility (*F*_1,147_ = 9.99, *p* = 0.01), crude protein (*F*_1,147_ = 11.96, *p* < 0.001), acid detergent fibre (*F*_1,147_ = 4.06, *p* = 0.03), total phenolics (*F*_1,147_ = 6.96, *p* = 0.004) and condensed tannins (*F*_1,147_ = 6.27, *p* = 0.006, but not neutral detergent fibre (*F*_1,147_ = 3.09, *p* = 0.06). Species had a significant effect on leaf dry matter digestibility (*F*_1,147_ = 12.00, *p* < 0.01), crude protein (*F*_1,147_ = 11.34, *p* < 0.001), neutral detergent fibre (*F*_1,147_ = 53.13, *p* < 0.001), acid detergent fibre (*F*_1,147_ = 4.56, *p* < 0.05), total phenolics (*F*_1,147_ = 13.72, *p* < 0.01) and condensed tannins (*F*_1,147_ = 70.60, *p* < 0.001). We identified fifteen woody species in the rangeland (Table 1), with five mostly foraged on by goats (three fine-leaved and two broad-leaved). The latter species were the most abundant.

**Table 1:** Percent abundance of the major browse species and leaf type.

Browse species	%	Leaf type
*Dichrostachys cinerea*	22.27	fine
*Acacia karroo*	19.94	fine
*Acacia nilotica*	18.66	fine
*Searsia (Rhus) tenuinervis*	9.97	broad
*Ziziphus mucronata*	8.45	broad
*Brachystegia spiciformis*	5.32	broad
*Strychos spinosa*	4.56	broad
*Gymnosporia senegalensis*	2.28	broad
*Combretum collinum*	1.85	broad
*Euclea divinorum*	1.28	broad
*Terminalia sericea*	1.23	broad
*Flacourtia indica*	1.23	broad
*Azanza garckeana*	1.14	broad
*Lannea discolor*	1.09	broad
*Piliostigma thonningii*	0.71	broad

*Ziziphus mucronata* and *S*. *tenuinervis* (broad-leaved) had higher Jacob’s selectivity index than the fine-leaved (*A*. *nilotica*, *A*. *karroo* and *D*. *cinerea*) in wet (both early and late wet) season in 2014 and 2015 (Table 2). During dry season the trend was reversed with fine-leaved (except for *A*. *nilotica* in 2014) having higher Jacob’s selectivity index than broad-leaved species. The Jacob’s selectivity index was significantly different between broad- and fine-leaved species (pooled data) (Table 3).

**Table 2:** Jacob’s selectivity index (*E*_*i*_) (mean ± S.E.) of five woody species browsed by goats in central Zimbabwe.

Browse species	2014				2015			
	Early wet season	Late wet season	Dry season	*F*_2,39_ -ratio	Early wet season	Late wet season	Dry season	*F*_2,39_ -ratio
*Ziziphus mucronata*	0.29 ± 0.01^Bb^	0.43 ± 0.01^Aa^	-0.10 ± 0.01^Cd^	823.72***	0.48 ± 0.01^Aa^	0.35 ± 0.01^Bb^	-0.20 ± 0.01^Cd^	1826***
*Searsia tenuinervis*	0.49 ± 0.02^Aa^	0.41 ± 0.00^Aa^	-0.22 ± 0.01^Be^	247.74***	0.42 ± 0.01^Ab^	0.37 ± 0.19^Ba^	-0.05 ± 0.00^Cc^	1656***
*Acacia nilotica*	-0.05 ± 0.00^Ac^	-0.06 ± 0.00^Ab^	-0.03 ± 0.00^Bc^	11.94***	-0.08 ± 0.00^Bc^	-0.03 ± 0.00^Bd^	0.12 ± 0.00^Ab^	698.41***
*Acacia karroo*	-0.07 ± 0.00^Bc^	-0.07 ± 0.00^Bb^	0.22 ± 0.01^Ab^	1178***	-0.07 ± 0.00^Bc^	-0.13 ± 0.00^Ce^	0.11 ± 0.00^Ac^	776.26***
*Dichrostachys cinerea*	-0.07 ± 0.00^Bc^	-0.06 ± 0.00^Bb^	0.26 ± 0.00^Aa^	2228***	-0.14 ± 0.00^Cd^	0.04 ± 0.00^Bc^	0.23 ± 0.00^Aa^	2194***
*F*_4,65_- ratio	739.01***	211.49***	1443***		1635***	2123***	1550***	

Means within the same browse species (A, B, C) and same season (a, b, c, d, e) followed by the same letter are not significantly different.

***p < 0.001. *Ziziphus mucronata* and *S*. *tenuinervis* were broad-leaved and *A*. *nilotica*, *A*. *karroo* and *D*. *cinerea* fine-leaved.

**Table 3:** Jacob’s selectivity index (*E*_*i*_) (mean ± S.E.) of broad- and fine-leaved woody species browsed by goats in central Zimbabwe.

Leaf type	2014				2015			
	Early wet season	Late wet season	Dry season	*F*_2,81_ -ratio	Early wet season	Late wet season	Dry season	*F*_2,81_ -ratio
Broad-leaved	0.39 ± 0.02^A^	0.42 ± 0.02^A^	-0.16 ± 0.01^B^	326.89***	0.45 ± 0.01^A^	0.36 ± 0.00^B^	-0.13 ± 0.02^C^	833.40***
Fine-leaved	-0.06 ± 0.00^B^	-0.06 ± 0.00^B^	0.15 ± 0.02^A^	103.47***	-0.10 ± 0.01^C^	-0.04 ± 0.00^B^	0.15 ± 0.01^A^	232.86***
*t-* test	21.25***	24.28***	-13.04***		50.10***	32.52***	-16.15***	

Means within the same leaf type (A, B, C) followed by the same letter are not significantly different.

***p < 0.001.

Dry matter digestibility and CP decreased from wet to dry season, while fibre (both NDF and ADF), TP and CT increased from wet to dry season in 2014 and 2015 (Table 4). *Acacia nilotica* and *Z*. *mucronata* were the most digestible in 2014 and 2015 respectively, while *D*. *cinerea* was the least digestible in the two years. In 2014, *A*. *karroo* and *D*. *cinerea* had the highest CP, while in 2015 *Z*. *mucronata* had the highest CP in the wet season. *Dichrostachys cinerea* was the most fibrous, that is, had highest NDF and ADF and also had the highest TP content.

**Table 4:** Dry matter digestibility (%) and leaf chemical composition (% dry matter) of five woody species browsed by goats in central Zimbabwe.

^Year Season^	^2014^				^2015^			
	Early wet season	Late wet season	Dry season	*F*_2,18_ -ratio	Early wet season	Late wet season	Dry season	*F*_2,18_ -ratio
***Ziziphus mucronata***								
DMD	75.31^A^	74.47^A^	63.27^B^	691.14***	73.29^B^	75.40^A^	60.85^C^	729.41***
CP	21.32^A^	20.24^B^	17.52^C^	50.93***	27.35^A^	25.09^B^	12.36^C^	1034***
NDF	29.11^C^	33.46^B^	38.25^A^	351.65***	34.33^C^	37.26^B^	43.44^A^	1599***
ADF	21.09^B^	20.75^B^	33.42^A^	1056***	25.83^B^	22.11^C^	33.71^A^	487.26***
TP	1.06^C^	4.38^B^	9.68^A^	1203***	4.96^C^	5.47^B^	8.35^A^	439.14***
CT	0.27^C^	0.56^B^	0.85^A^	157.20***	0.57^C^	0.61^B^	0.86^A^	176.74***
***Searsia tenuinervis***								
DMD	78.44^A^	70.41^B^	65.10^C^	588.90***	67.21^A^	60.25^C^	61.96^B^	189.59***
CP	25.23^A^	17.30^B^	11.47^C^	743.54***	20.25^A^	15.93^B^	10.28^C^	369.58***
NDF	27.52^C^	34.03^B^	38.09^A^	393.82***	35.02^C^	35.42^B^	39.18^A^	241.05***
ADF	19.12^C^	24.40^B^	27.45^A^	265.49***	30.11^C^	36.30^A^	30.56^B^	792.48***
TP	1.11^C^	1.96^B^	5.05^A^	1829***	3.21^B^	2.70^C^	5.98^A^	201.08***
CT	0.53^B^	0.79^A^	0.88^A^	53.49***	1.17^B^	0.98^C^	2.10^A^	93.79***
***Acacia nilotica***								
DMD	81.38^A^	70.38^B^	66.17^C^	939.17***	67.40^A^	67.18^A^	61.91^B^	125.50***
CP	21.11^A^	21.11^A^	15.30^B^	206.01***	17.27^B^	20.20^A^	15.08^C^	109.56***
NDF	20.01^C^	34.18^B^	38.88^A^	824.13***	34.44^C^	37.31^B^	38.55^A^	569.99***
ADF	13.34^C^	26.12^B^	29.09^A^	867.76***	28.11^C^	30.27^B^	34.63^A^	687.37***
TP	3.46^C^	5.00^B^	8.43^A^	133.69***	6.55^B^	5.68^C^	9.42^A^	286.26***
CT	0.06^C^	1.09^B^	2.86^A^	1114***	1.01^C^	1.15^B^	1.24^A^	54.33***
***Acacia karroo***								
DMD	76.39^A^	68.55^B^	64.26^C^	808.60***	69.17^A^	67.26^B^	64.39^C^	72.15***
CP	28.11^A^	22.15^B^	20.07^C^	347.52***	23.04^A^	20.17^B^	20.11^B^	50.63***
NDF	38.97^C^	41.09^B^	46.42^A^	228.86***	43.35^B^	42.28^B^	46.42^A^	86.44***
ADF	23.29^C^	29.27^B^	33.37^A^	382.21***	28.99^C^	29.40^B^	33.18^A^	211.94***
TP	0.24^C^	1.77^B^	3.65^A^	238.40***	1.53^B^	1.53^B^	2.65^A^	77.67***
CT	0.26^C^	0.36^B^	0.90^A^	284.35***	0.45^C^	0.48^B^	0.62^A^	31.29***
***Dichrostachys cinerea***								
DMD	67.43^A^	61.51^B^	57.50^C^	471.99***	63.26^B^	64.33^A^	60.24^C^	69.08***
CP	28.10^A^	22.22^B^	19.81^C^	318.04***	25.16^A^	25.17^A^	19.39^B^	178.32***
NDF	45.04^C^	48.22^B^	56.08^A^	480.46***	51.22^B^	49.35^C^	57.03^A^	332.25***
ADF	33.27^C^	38.63^B^	41.16^A^	229.92***	37.13^B^	35.53^C^	38.19^A^	29.16***
TP	4.59^C^	6.52^B^	9.36^A^	159.23***	6.94^C^	7.71^B^	9.47^A^	501.69***
CT	0.24^C^	0.38^B^	1.35^A^	572.80***	0.83^B^	1.05^A^	1.15^A^	232.00***

Means in the same row (within each year) followed by the same letter are not significantly different. DMD: Dry matter digestibility, CP: Crude protein, NDF: Neutral detergent fiber, ADF: Acid detergent fiber, TP: Total phenolics, CT: Condensed tannins.

***p < 0.001.

Leaf type had no effect on dry matter digestibility (broad-leaved: 68.83 ± 1.87 vs fine-leaved: 66.60 ± 1.33, Independent *t-test* = 0.97, *p* = 0.34) and leaf chemical composition (CP–broad-leaved: 18.70 ± 1.62 vs fine-leaved: 21.31 ± 0.87, Independent *t*-test = -1.43, *p* = 0.17; ADF—broad-leaved: 27.07 ± 1.66 vs fine-leaved: 31.28 ± 1.53, Independent *t*-test = -1.86, *p* = 0.07; TP—broad-leaved: 4.49 ± 0.78 vs fine-leaved: 5.25 ± 0.71, Independent *t*-test = -0.72, *p* = 0.48; CT–broad-leaved: 0.85 ± 0.13 vs fine-leaved: 0.86 ± 0.15, Independent *t*-test = -0.06, *p* = 0.95), except NDF (broad-leaved: 35.43 ± 1.25 vs fine-leaved: 42.71 ± 2.06, Independent *t*-test = -3.02, *p* = 0.006).

## Discussion

Our results were in support of the first hypothesis that free-ranging goats select broad-leaved more than fine-leaved woody species during wet (growth) season and fine-leaved more than broad-leaved woody species during dry season. This means that goats switched from broad-leaved *Z*. *mucronata* and *S*. *tenuinervis* to fine-leaved *A*. *nilotica*, *A*. *karroo* and *D*. *cinerea* between wet and dry season presumably in response to changes in shoot and leaf availability. Broad-leaved woody species were sprouting and had plenty of foliage during wet season but shed their leaves in the dry season, while fine-leaved species retained their leaves well into the dry season (Nyamukanza personal observations). This implies that when both broad- and fine-leaved woody species have plentiful foliage, goats will select broad- over fine-leaved, only switching to fine-leaved when broad-leaved species have lost most of their foliage. The higher selection of broad-leaved than fine-leaved species during wet season was presumably due to their high foliar mass [4]. The switch from broad- to fine-leaved woody species between wet and dry season shows the plasticity of the foraging behaviour of goats in response to changes in foliage availability. Mellado [5] suggested that goat flexibility in browse choice was aimed at achieving feed intake of approximately 3% of body weight of highly digestible diets for most of the year to meet their nutritional requirements. Goats are able to adapt to seasonal changes in browse availability and quality [5,42]. In agreement with our findings, Mellado [5] reported goats as selecting fine-leaved woody species more than broad-leaved species during dry season and broad-leaved over fine-leaved during early wet season. Goats tend to select deciduous woody species that retain leaves well into the dry season [4]. Schroeder et al. [43] reported goats browse selection as influenced by foliage availability.

Woody species abundance could have also influenced goat browse foraging choices as the five most browsed species were the most abundant, although not in the order of abundance [5,44,45]. Consistent with findings by Mellado [5] that inter-annual variation does not affect goat foraging choices our results were similar for 2014 and 2015.

Our findings did not support the second hypothesis that broad- and fine-leaved woody species have different foliar dry matter digestibility and chemical composition. This implies that other factors and not nutritive value were responsible for the varying selection of broad- and fine-leaved woody species with the most plausible explanation being that foliar availability variations between wet and dry season influenced goat browse choices. From a nutritional standpoint both broad- and fine-leaved woody species are good nutrient sources, although feeding trials are required to ascertain their utilization by goats after consumption. Basha et al. [6] also reported leaf chemical composition as not explaining goat browse selection. Mellado [5] argued that it was not clear which physical features and chemical compounds were used by goats to select nutritive browse.

Consistent with other studies browse quality was high (high DMD and CP, low fibre, TP and CT) during wet season and decreased in the dry season [10,18,46]. The decrease in dry matter digestibility and increase in fibre content (both NDF and ADF) in all the woody species from wet to dry season means that goats consume foliage of low nutritive value during the dry season negatively affecting their productivity. Fibre (NDF and ADF) is negatively correlated to dry matter digestibility [47]. The DMD of 60.24–81.38% during the year was comparable to that of 44–65% reported to be of forage selected by goats [5]. The CP ranged from 10.28% to 28.11% compared to 8–16% for forage selected by goats [5]. Forage with a CP content of 10–13% and 50–70% DMD is considered to be highly nutritious [5]. A dietary CP requirement for goat maintenance, pregnancy and lactation is 13% [48]. The NDF ranged from 20.01 to 57.03% compared to 43–54% reported by Mellado [5]. The increase in fibre content from wet to dry season was because leaves were young and tender during wet season and then became mature and fibrous during dry season. Both NDF and ADF increase with leaf maturity [49]. Thus, during the dry season goats forage on mature leaves with high fibre content. For example, Manousidis et al. [50] reported a strong positive effect of NDF on diet selection during dry season.

The increase in TP from wet to dry season was in agreement with findings by Martz et al. [51] that plant secondary metabolite concentrations are low in the growing (wet) season.

## Conclusion

We conclude that goats showed high plasticity in browse selection between broad- and fine-leaved species presumably driven by availability of foliage. During wet season when browse was generally plentiful the goats selected broad-leaved species presumably to increase foliar intake and then switched to fine-leaved woody species which retained green leaves during dry season. In addition, leaf digestibility and chemical composition was similar between broad- and fine-leaved woody species throughout the year.

## Supporting information

S1 Dataset Species selection. (XLSX)

S2 Dataset Species abundance. (XLSX)
